# Adenosine A_2A_ receptor activation reduces recurrence and mortality from *Clostridium difficile* infection in mice following vancomycin treatment

**DOI:** 10.1186/1471-2334-12-342

**Published:** 2012-12-10

**Authors:** Yuesheng Li, Robert A Figler, Glynis Kolling, Tara C Bracken, Jayson Rieger, Ralph W Stevenson, Joel Linden, Richard L Guerrant, Cirle Alcantara Warren

**Affiliations:** 1 Division of Infectious Diseases and International Health, Carter Harrison Bldg, MR6-Rm 2705, 345 Crispell Drive, Charlottesville, VA 22908, USA; 2 HemoShear, LLC, Charlottesville, VA, 22908, USA; 3 Human Therapeutics Division, Intrexon Corporation, Germantown, MD, 20876, USA; 4 Dogwood Pharmaceuticals, Inc., New Haven, CT, 06511, USA; 5 Division of Inflammation Biology, La Jolla Institute for Allergy & Immunology, La Jolla, CA, 92037, USA; 6 University of Virginia, Charlottesville, VA, 22908, USA

**Keywords:** *C*. *difficile*, Colitis, Adenosine A_2A_ receptor, Diarrhea

## Abstract

**Background:**

Activation of the A_2A_ adenosine receptor (A_2A_AR) decreases production of inflammatory cytokines, prevents *C*. *difficile* toxin A-induced enteritis and, in combination with antibiotics, increases survival from sepsis in mice. We investigated whether A_2A_AR activation improves and A_2A_AR deletion worsens outcomes in a murine model of *C*. *difficile* (strain VPI10463) infection (CDI).

**Methods:**

C57BL/6 mice were pretreated with an antibiotic cocktail prior to infection and then treated with vancomycin with or without an A_2A_AR agonist. A_2A_AR^-/-^ and littermate wild-type (WT) mice were similarly infected, and IFNγ and TNFα were measured at peak of and recovery from infection.

**Results:**

Infected, untreated mice rapidly lost weight, developed diarrhea, and had mortality rates of 50-60%. Infected mice treated with vancomycin had less weight loss and diarrhea during antibiotic treatment but mortality increased to near 100% after discontinuation of antibiotics. Infected mice treated with both vancomycin and an A_2A_AR agonist, either ATL370 or ATL1222, had minimal weight loss and better long-term survival than mice treated with vancomycin alone. A_2A_AR KO mice were more susceptible than WT mice to death from CDI. Increases in cecal IFNγ and blood TNFα were pronounced in the absence of A_2A_ARs.

**Conclusion:**

In a murine model of CDI, vancomycin treatment resulted in reduced weight loss and diarrhea during acute infection, but high recurrence and late-onset death, with overall mortality being worse than untreated infected controls. The administration of vancomycin plus an A_2A_AR agonist reduced inflammation and improved survival rates, suggesting a possible benefit of A_2A_AR agonists in the management of CDI to prevent recurrent disease.

## Background

*Clostridium difficile* infection (CDI) is characterized by intense intestinal and systemic inflammatory reactions, especially in moderate to severe disease. Such microorganism-initiated tissue damage causes *de novo* production and *in situ* accumulation of adenosine that signals through four G protein–coupled receptors designated as A_1_, A_2A_, A_2B_, and A_3_[1]. Activation of the A_2A_ adenosine receptor (A_2A_AR) produces a constellation of responses that are anti-inflammatory. Pro-inflammatory responses in bone marrow derived cells (BMDC) including platelets [2], monocytes [3], mast cells [4,5], neutrophils [6-8] and T cells [9-11] are all inhibited by A_2A_AR activation.

Adenosine is a purine nucleoside that plays an important role in many biochemical processes such as energy transfer. It also acts as a secondary messenger and neurotransmitter [12]. Endogenous adenosine is produced in part by nucleotide degradation with participation of 5^′^ nucleotidase with or without ectonucleotidases on cell membranes, from the pool of adenosine triphosphate (ATP), adenosine diphosphate (ADP), or adenosine monophosphate (AMP) released through regulatory processes, inflammation, or cellular damage. Adenosine can further be degraded to inosine by adenosine deaminase intra- and/or extra-cellularly. Otherwise, it can be retaken up and converted back to AMP by adenosine kinase. As an endogenous purine nucleotide, adenosine modulates many physiological processes through four adenosine receptor subtypes, A_1_, A_2A_, A_2B_ and A_3_.

Selective activation of the A_2A_AR with synthetic adenosine analogs has been demonstrated to protect many tissues, including liver, kidney, skin, heart, and spinal cord, from ischemia-reperfusion injury [13-16], to inhibit inflammatory responses in rabbit joint sepsis induced by LPS [17], and to improve mouse survival from sepsis with *Escherichia* coli [18,19] or *Staphylococcus* aureus [18] in combination with antibiotic treatment. Previous studies have suggested that activation of A_2A_ARs with ATL 313, or inhibition of adenosine deaminase prevents *Clostridium difficile* toxin A-induced enteritis by reducing the production of inflammatory cytokines in mouse or rabbit ileal loop model [20-22]. In the current study, we found that A_2A_AR activation during antibiotic treatment for CDI lessens disease severity, prevents relapse and increases survival of mice. Deletion of A_2A_ARs worsens outcome of CDI by enhancing the host inflammatory response to infection. The beneficial effects of A_2A_R activation are probably caused by anti-inflammatory effects of A_2A_AR activation counteracting the pro-inflammatory effects of *C*. *difficile* toxins.

## Methods

### Animals

Eight-week old male C57BL/6 mice were purchased from the Jackson Laboratory (Bar Harbor, ME 04609). Food and water were provided *ad libitum* before and during the experiments. A_2A_AR -/- mice from Jiang-Fan Chen [23] of Boston University were bred to be congenic with C57BL/6 mice. A_2A_AR -/- mice were age- and sex-matched to wild type controls. Mouse genotyping employed a set of 3 primers (5^′^-GGGCTCCTCGGTGTACAT-3^′^, 5^′^-CCCACAGATCTAGCCTTA-3^′^, 5^′^-TGTCACGTCCTGCACGAC-3^′^) to resolve a 380-bp wild type allele versus a 500-bp knockout allele. Animals were housed in a pathogen-free isolation barrier facility with chip bedding. A previously published infection model was adapted with slight modification [24]. Briefly, all mice were started with a 3-day antibiotic cocktail pretreatment containing 4.5 mg of vancomycin, 4.2 units of colistin, 3.5 mg of gentamicin, and 21.5 mg of metronidazole per kg/day in drinking water 6 days before the infection. Clindamycin (32 mg/kg) was given intraperitoneally to each mouse the day before the infection. Mice were transferred from a pathogen-free room to a BSL-2 room within the vivarium where they were prepared for infection. Infected mice remained in the same cage and were placed in a dedicated sash in the BSL-2 room. In most experiments, 50 mg/kg/day of vancomycin was administered in drinking water starting 24 hours post infection. The vancomycin treatment was routinely terminated on day 4 post infection unless specifically stated. Treatment with an A_2A_AR agonist (via Alzet pump as described below) was typically started 1 day post-infection (unless specified) and lasted for either 7 or 14 days. Mice that were considered moribund were euthanized by cervical dislocation. Infection and treatment protocol was approved by the University of Virginia Animal Care and Use Committee.

### Materials

**Figure 1:**
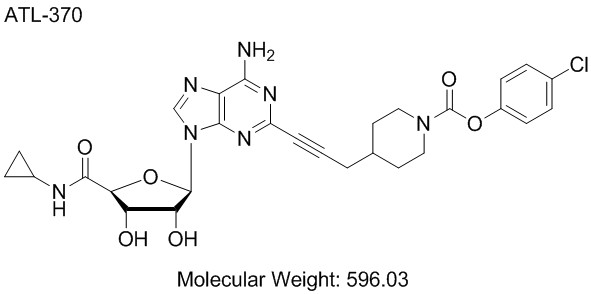
**Molecular Structure and Weight of A**_**2A**_**AR Agonist**, **ATL370**.

The A_2A_AR agonists ATL370 and ATL1222 as well as ALZET mini-osmotic pumps (Model 1007D and 1002) were gifts from Dogwood Pharmaceuticals, Inc (Charlottesville, VA). The chemical structure and molecular weight of ATL370 are shown in Figure 1. The molecular weight of the ATL370 analogue, ATL1222, is 539.25 and its potency at the adenosine receptor subtypes is similar to ATL370. Injectable vancomycin hydrochloride (Hospira, Inc., Lake Forest, IL), Colistimethate (Colistin) (X-GEN Pharmaceuticals, Inc., Big Flats, NY), gentamicin sulfate (Hospira), metronidazole (Flagyl) (Baxter Healthcare Corporation, Deerfield, IL), and cleocin phosphate (clindamycin) (Pharmacia and Upjohn Company, Bridgewater, NJ) were all purchased through the University of Virginia Hospital Pharmacy. *C*. *difficile* strain VPI10463 was purchased from American Type Culture Collection (Manassas, VA). Chopped Meat Broth (CM, catalog AS-811) for *Clostridium difficile* growth was purchased from Anaerobe Systems (Morgan Hill, CA). Bacto Brain Heart Infusion (BHI medium) for *C*. *difficile* wash and reconstitution was purchased from BD Medical-Pharmaceutical Systems (Franklin Lakes, NJ). Bouin’s fixative was purchased from Polysciences, Inc. (Warrington, PA). QIAamp DNA Stool Mini Kit and Proteinase K were purchased from Qiagen, Inc. (Valencia, CA). iQ SYBR Green Supermix containing dNTPs, iTaq DNA polymerase, 6mM MgCl_2_, SYBR Green I, fluorescein, and stabilizers was obtained from Bio-Rad Laboratories (Hercules, CA). All PCR primers were ordered through Integrated DNA Technologies, Inc. (Coralville, IA).

### Radioligand binding assays

The adenosine receptor binding assay methodology has been described previously [8] and was conducted by Dogwood Pharmaceuticals (Charlottesville, VA 22911). In brief, all subtypes of recombinant human and mouse ARs were stably expressed in HEK-293 cells (A_2A_AR, A_2B_AR, and A_3_AR), CHO-K1 cells (A_1_AR) or transiently expressed by baculoviral infection of Sf9 cells (A_2A_AR, high-affinity assay). Crude membranes were prepared from these transfected cells. An appropriate radioligand (^125^I-ABA for A_1_AR and A_3_AR, ^125^I-ZM241385 for A_2A_AR, ^125^I-ABOPX for A_2B_ AR, or ^125^I-APE for A_2A_AR/high affinity assay) was added, then filtered through glass fiber filters, and counted in a Wallac Wizard 1470 gamma counter (Perkin Elmer, Boston MA). The non-specific binding of radiolabeled ligand was measured in the presence of the non-selective AR agonist, NECA (100 μM). G protein coupled receptors bound agonists with two affinity states (G protein coupled and G protein uncoupled). The coupled high affinity state was the more relevant assay because it reflects the active configuration of the receptor. To produce G protein coupled human A_2A_ARs, Sf9 cells were simultaneously infected with 4 baculoviruses encoding the human A_2A_AR and G protein α_s_, β_4_ and γ_2_ subunits [25]. High affinity binding was performed using ^125^I-APE as described above. Competition binding curves were constructed and IC_50_ values calculated using a 4-parameter logistic fit (PRISM 5.0, GraphPad Software, San Diego, CA). The value of *K*_i_ for competition for radioligand binding by agonist was calculated using the Cheng-Prusoff equation [26]. *K*_i_ values were calculated from the mean of triplicate or quadruplicate assays and normalized to the mean value of the inter-assay standard.

### Preparation of *Clostridium difficile* inocula

*C*. *difficile* was grown for 20 hours in CMB (37°C), transferred to a fresh tube of CMB and incubated for 5 hours to achieve log-phase growth prior to infection. Bacteria were centrifuged at 10,000 rpm for 2 minutes, washed with BHI medium three times, reconstituted with BHI medium, and quantified by spectrophotometry. An OD reading of 1.0 was calculated to be equivalent to 5×10^7^ cfu *C*. *difficile*/ml. The final concentration of *C*. *difficile* (cfu/ml) was validated by hemocytometer reading under light microscopy. Unless the dose was specifically stated, 1×10^4-5^ of *C*. *difficile* was gavaged to each mouse in the infected groups, and BHI medium only was given to mice in uninfected groups.

### Implantation of ALZET osmotic pumps

ALZET mini-osmotic pumps (Model 1007D and 1002) were implanted in mice using a protocol approved by the University of Virginia Animal Care and Use Committee. Briefly, mice were anesthetized with a mixture of ketamine and xylazine and placed on a heating pad. A short (~5 mm) incision was made at the interscapular area after shaving and cleaning the skin with 70% ethanol and iodine. Pumps filled with A_2A_AR agonists (ATL370 or ATL1222 at either 1 or 10 ng/kg/min) for treated mice or an equivalent amount of the vehicle-3% dimethyl sulfoxide (DMSO) in PBS only, for untreated control mice were inserted subcutaneously. A wound clip was used to close the incision. Mice were allowed to wake up on the heating pad before returning to the BSL2 sash.

### Clinical scoring system

During the entire post infection period, daily weights and clinical scores were recorded for each mouse (Table 1). The sum of all parameter scores was considered the final clinical score and ranged from 0 (normal) to 15. Mice judged to be moribund were sacrificed. Mice found dead were assigned a score of 15. Stools were collected daily and stored at -20°C until DNA extraction was performed.

**Table 1:** **Clinical scoring system for mice infected with *Clostridium difficile***

**Category**	Scores*			
	**0**	**1**	**2**	**3**
***Activity***	**normal**	**Alert**/**slow moving**	**Lethargic**/**shaky**	**Inactive unless prodded**
***Posture***	**normal**	**Back slanted**	**Hunched**	**Hunched**/**nose down**
***Coat***	**normal**	**Piloerection**	**Rough skin**	**Very ruffled**/**puff**/ **Ungroomed**
***Diarrhea***	**normal**	**Soft stool**/**discolored** (**yellowish**)	**Wet stained tail**/ **mucous** +/- **blood**	**Liquid**/**no stool** (**ileus**)
***Eyes***/***Nose***	**normal**	**Squinted** ½ **closed**	**Squinted**/**discharge**	**Closed**/**discharge**

*Clinical Score=sum of all parameter scores. Total possible score=15. Normal=0; Found dead=15.

### Extraction of DNA from frozen mouse stools

All stool samples were weighed before DNA extraction for sample normalization. Stool DNA was extracted under the modified protocol provided in the QIAamp DNA Stool Mini Kit. Briefly, frozen stool was added to 400 μL of ASL buffer, homogenized by grinding with a wooden stick, vortexed for 15 seconds before and after heating in a water bath at 82.5°C for 5 minutes, and then centrifuged at 14,000 rpm for 2 minutes. The remaining steps followed the manufacturer’s directions. Extracted stool DNA was stored at -20°C prior to PCR testing.

### Quantification of *C*. *difficile* shedding by qPCR

*C*. *difficile* DNA was analyzed from extracted stool DNAs with iQ SYBR Green Supermix in a 96-well plate performed at CFX96™ Real-Time PCR Detection System (Bio-Rad). Briefly, a PCR master mix was prepared with 23 μL aliquot containing 1 μL each of *tcdB* forward and reverse primers, 12.5 μL iQ SYBR Green Supermix, and 8.5 μL of H_2_O purified by Milli-Q Integral Water Purification System (Millipore Corporation, Billerica, MA). 2 μL of each sample was then added to each well filled with PCR master mix aliquot in 96-well. The PCR parameters were sequentially set for 3 stages: 1× cycle for 5 minutes at 94.0°C, 40 × cycle for 30 seconds each from 94.0°C and 55.0°C to72.0°C, 64 × cycle at 62.0°C for 15 seconds, and 1× cycle for hold at 25.0°C. Melt curve data collection and analysis were enabled. Copy numbers of unknown sample were extrapolated from the standard curve that was generated with the extracted DNA prepared from known *C*. *difficile* inocula. The sequence of *tcdB* forward primer was 5^′^-GGAGAGTCATCCAACTTATATG-3^′^; the sequence of *tcdB* reverse primer was 5^′^-CCACCAATTTCTTTTAATGCAG-3^′^.

### Intestinal histopathology

The middle cross section of cecum and proximal cross section of colon were harvested from moribund mice or surviving mice at the end of the experiment. Tissues were fixed overnight with Bouin’s solution and stored in 70% ethanol until subsequently processed for Hematoxylin & Eosin (HE) staining at University of Virginia Research Histology Core. Slides were examined using a Leica DFC425 digital camera equipped microscope with Leica Application Suite Version 3.6.0.488 imaging software (Leica Microsystems Inc., Buffalo Grove, IL 60089). Intestinal tissues were scored from 0 to 3 (with zero as normal and 3 as the worst pathologic score) under 5 categories: overall architecture, mucosal thickness, submucosal edema, inflammation, and exudates.

### ELISA for IFNγ and TNFα

One set of infection experiments with A_2A_AR -/- (n=8) and their littermate A_2A_AR +/+ (n=8) mice were set aside for blood and tissue cytokine assays at the peak of infection (day 3) and also at recovery (day 7). Blood samples were collected in heparinized tube by cardiopuncture under sedation. Plasma samples were obtained by centrifuging blood at 10,000 rpm at room temperature for 20 minutes and stored at -80°C until further analysis. Upon euthanasia, cecal samples were harvested and stored at -80°C. ELISA was performed using Thermo Scientific Pierce Mouse IFNγ and TNFα kits (Rockford, IL) with slight modification of the manufacturer’s instruction. Briefly, cecal tissue was homogenized by grinding on dry ice and suspended in diluent reagent (0.2 mg/ml). Each homogenate or plasma sample was incubated with IFNγ antibody (for 1 hour) or TNFα antibody (for 2 hours) in 1:20 final dilution (200 μl) at room temperature. After washing, the sample was incubated with 100 μl of streptavidin-horseradish peroxidase (HRP) for 30 minutes. Subsequently, 100 μl of TMB substrate was added for another 30-minute incubation in the dark. The incubation was terminated with 100 μl of stop solution. Samples were immediately measured at 450 nm and 550 nm in Gen5 1.11.5 version in BioTek Spectrophotometer (Winooski, Vermont). Standard curves were established by plotting the average absorbance obtained for each standard known concentration (pg/ml). Cytokine amount in each sample was extrapolated from the standard curves.

### Statistical analysis

Statistical analyses were conducted using GraphPad Prism Version 5.02 software. When mouse was either found dead or sacrificed due to severe distress, its last body weight recording was continuously plotted against the body weights of surviving mice. Differences between groups for the entire experimental period were analyzed by 2-Way ANOVA with Bonferroni post hoc testing. Survival curves were analyzed using Log-rank (Mantel-Cox) or Log-rank test for mortality trend.

## Results

### A_2A_AR agonist reduced diarrhea and deaths in *C*. *difficile*- infected mice

**Figure 2:**
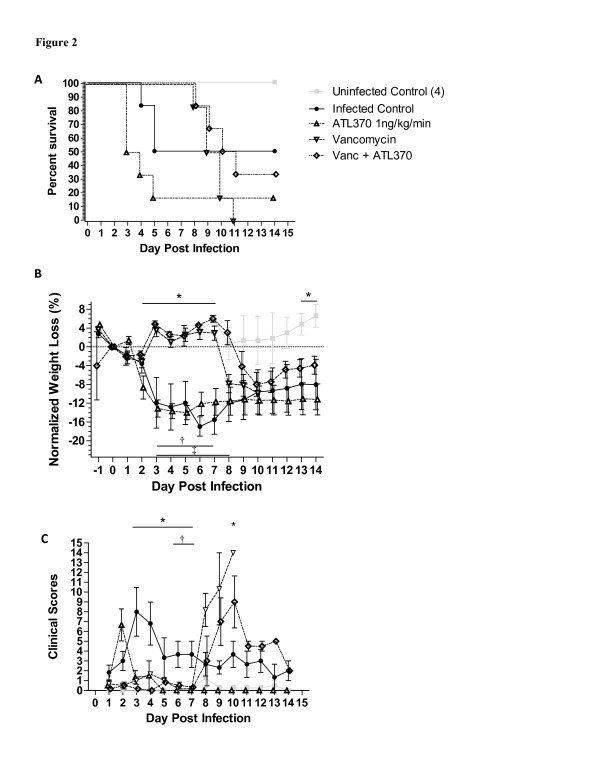
**Effect of A**_**2A**_**AR Agonist ATL370 on *C***. ***difficile***-**Infected Mice.** Mice were infected with VPI10463 at 10^5^ inoculum and treated or not with vancomycin (50 mg/kg/day for 3 days) with or without ATL370 (1 ng/kg/min). Each group had 6 mice each except for uninfected control (n=4). For statistical analyses of weight change and clinical scores, the latest value from a dead mouse was carried through to the end of observation but not shown in the graphs. **A**. Survival curve. P=0.0016 by Mantel-Cox. **B**. Percent weight loss from day 0 (day of infection). *p<0.05 to 0.001 for uninfected (UC) vs. infected (I) controls, ^†^p<0.01 to 0.001 for I vs I with vancomycin (Vanc), ^‡^p<0.01 to 0.001 for I vs. I with Vanc plus ATL370(1); by Two-way ANOVA with Bonferroni’s correction. **C**. Clinical scores. *p<0.05 for UC vs. I controls, ^†^p<0.05 for I vs I with Vanc; by Two-way ANOVA with Bonferroni’s correction.

To confirm the protective effect of A_2A_AR activation previously seen in ileal loop models [20-22], we used the A_2A_AR agonist ATL370 to treat wild-type mice infected with *C*. *difficile* VPI10463. The binding affinity of ATL 370 to adenosine receptors is shown in Table 2. Two dosing regimens were tested: ATL370 at 1 ng/kg/min via a 14-day (first study) and at 10 ng/kg/min via a 7-day (2^nd^ study) Alzet pumps. Mortality rate from infected controls in the first study was 50%, with deaths occurring at days 4 and 5 (Figure 2A). All mice treated with vancomycin alone had relapsed and succumbed to infection by day 11 (deaths occurred 5 to 11 days after discontinuation of the antibiotic). In contrast, survival increased to 33% in mice treated with both vancomycin and ATL30. From day 1 post infection, infected mice progressively lost weights with a few losing up to almost 20% of their body weight at baseline (Figure 2B). Infected mice treated with vancomycin did not have weight loss until four days after termination of vancomycin treatment. Infected mice treated with both vancomycin and ATL370 at 1 ng/kg/min lost the same amount of weight as the vancomycin only-treated mice although weight loss started one day later. Infected control mice had rapid development of diarrhea and elevated clinical scores (Figure 2C), whereas treatment with vancomycin prevented signs of disease until three days after termination of treatment. Consistent with weight changes, clinical symptoms tended to be lessened in mice receiving combination treatment compared to those treated with vancomycin alone. Clostridial shedding in the stool was elevated in infected mice not given vancomycin during acute infection (data not shown). Upon discontinuation of vancomycin, clostridial shedding increased significantly and remained elevated with or without ATL370 in surviving mice indicating that the agonist does not affect fecal clostridial burden. This experiment suggested a modest benefit of the A_2A_AR agonist ATL370 at 1 ng/kg/min if given with vancomycin during *C*. *difficile* infection. Furthermore, this study suggested that A_2A_AR activation during infection is detrimental in the absence of antibiotics.

**Table 2:** **Characterization of the A**_**2A**_**AR agonist**, **ATL370**, **by radioligand binding** (**mean** ± **SD**)

**Receptor**	**A**_**1**_**AR**	**A**_**2A**_**AR - high**^**a**^	**A**_**2A**_**AR- low**^**a**^	**A**_**2B**_**AR**	**A**_**3**_**AR**
Radioligand	^125^I-ABA	^125^I-APE	^125^I-ZM241385	^125^I-ABOPX	^125^I-ABA
Human (*K*_*i*_, nM)	61.1 ± 11.3	0.5 ± 0.2	3.8 ± 0.7	>10,000	130.4 ± 44.4
Mouse (*K*_*i*_, nM)	65.3 ± 15.8	N/A	4.3 ± 2.8	>10,000	84.2 ± 19.2

^a^Agonists are known to bind to two affinity states of G-protein coupled receptors. Low affinity reflects binding to uncoupled receptors. High affinity refers to binding to receptor-G protein complexes.

We next tested the effect of ATL370 at a higher dose but shorter duration of treatment, we used 10 ng/kg/min dosing given for 7 days (via Alzet pump). Similar to what was observed earlier, infected mice treated with vancomycin fared well for several days until discontinuation of vancomycin. Addition of ATL370 to vancomycin improved survival by 20%. Infected mice that received ATL370 alone had a 0% survival rate. Weight changes and clinical scores followed the same trend as the first study. Taken together, these data suggested that low dose ATL370 may be as beneficial as higher doses if given with vancomycin and confirmed that A_2A_AR agonist alone could exacerbate disease during acute infection.

### Early or delayed administration of A_2A_AR agonist improved outcome of *C*. *difficile* infection

**Figure 3:**
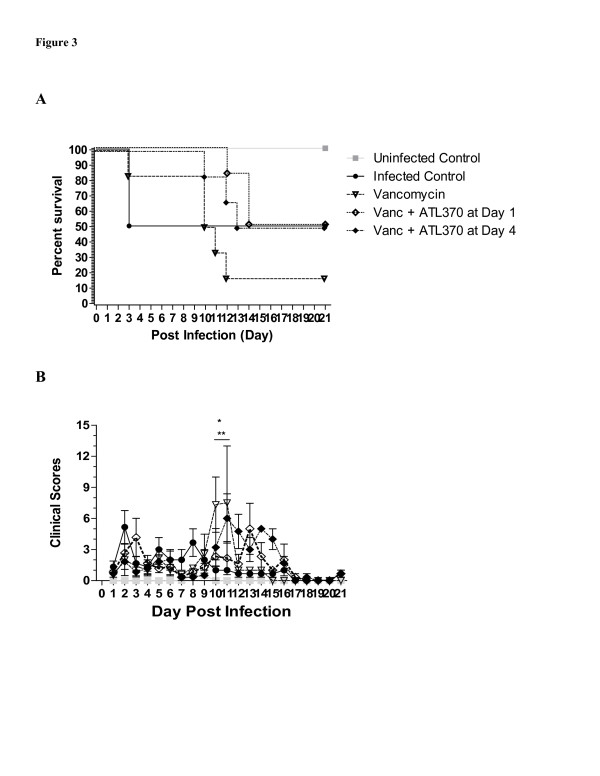
**Effect of Timing ofA**_**2A**_**ARAgonist ATL370 Treatment on *C ***.***difficile ***-**Infected Mice.** Mice were infected with VPI10463 at 10^5^ inoculum and treated or not with vancomycin (50 mg/kg/day for 3 days) with or without ATL370 (1 ng/kg/min started either at day 1 or day 4 after infection). **A**. Survival Curve, p=0.1427 by Mantel-Cox. **B**. Clinical Scores; ^**^p<0.01 for I vs. I with Vanc, ^*^p<0.05 to 0.01 for I vs. I with Vanc plus ATL370 by Two-way ANOVA.

To determine whether the timing of A_2A_AR agonist treatment would alter the response to treatment, we investigated the effect of delaying administration of ATL370 in relation to vancomycin. During the 21-day post infection period (Figure 3), we observed a 50% survival rate in the infected control group while those treated with vancomycin alone had only a 17% survival rate. Again, mortality in the vancomycin-treated mice was evident only after the antibiotic was discontinued suggesting relapse of infection. Treatment with ATL370, regardless of whether it was started at the same time as or 3 days after vancomycin was started, increased survival by 33%, indicating a benefit of A_2A_AR activation even at a later timepoint after infection or antibiotic treatment. Body weight change, diarrhea and clinical scores followed similar trends as shown in previous experiments.

### Reduced vancomycin exposure further increased survival in the presence of A_2A_AR agonist in mice infected with *C*. *difficile*

**Figure 4:**
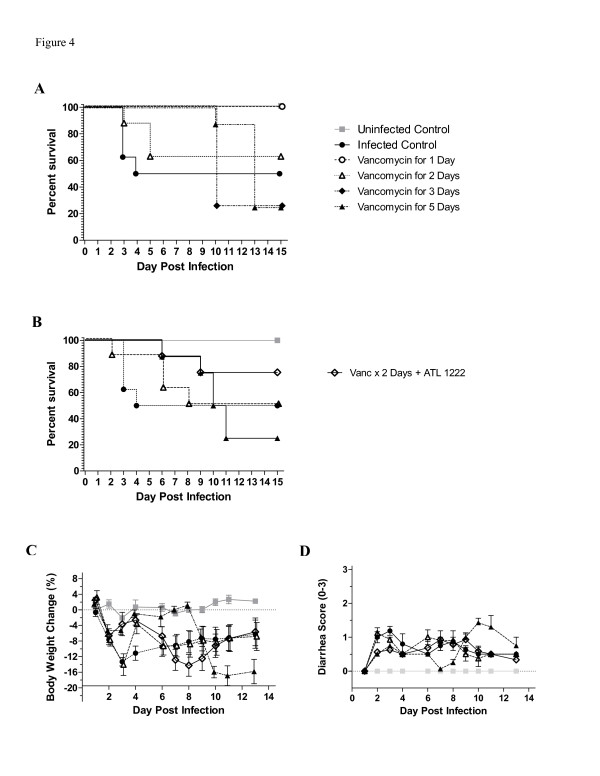
**Effect of A**_**2A**_**AR Agonist ATL1222 on Mortality of Mice Infected with *C ***.***difficile *and Treated with Vancomycin of Short or Long Duration.** Mice were infected with VPI10463 at 10^5^ inoculum. Each group had 8 mice except uninfected control (n=4). **A**. Mice were treated or not with vancomycin (50 mg/kg/day) for 1, 2, 3 or 5 days. P=0.058 by Log-rank (Mantel-Cox) Test. **B**. Mice were treated with vancomycin for either 2 or 5 days with or without ATL1222 (1ng/kg/min started at day 1 post-infection). *P* value for Log-rank (Mantel-Cox) test at 0.29. **C**. Weight Change (%). **D**. Diarrhea score (mean). Scoring system defined in Table 1.

Given that vancomycin administration had consistently resulted in delayed and worse mortality compared to untreated infection, we investigated whether decreasing duration of vancomycin treatment would improve survival and, therefore, enhance benefit derived from A_2A_AR activation. As shown in Figure 4A, short treatment durations (1-2 days) prevented recurrence of infection and significantly improved survival rate from 37.5% in longer treatment durations (3-5 days) to 87.5%. We, then, compared a short course (2-day) versus a long course (5-day) vancomycin treatment in combination with another A_2A_AR agonist, ATL1222. As shown in Figure 4B, infected mice treated with a 2-day treatment course had better survival rates than mice treated with a 5-day course (50% vs. 25%) of vancomycin. Furthermore, ATL1222 improved survival by 25% when given in addition to a 2-day course of vancomycin or by 50% when compared to a 5-day course of vancomycin alone. Both weights and diarrhea scores were likewise improved with the shorter course of antibiotics plus ATL1222 (Figure C&D). Together, these findings suggest that A_2A_AR activation enhances the benefit of a shorter course of antibiotic treatment against CDI.

### The absence of A_2A_AR worsened *C*. *difficile* infection in mice

**Figure 5:**
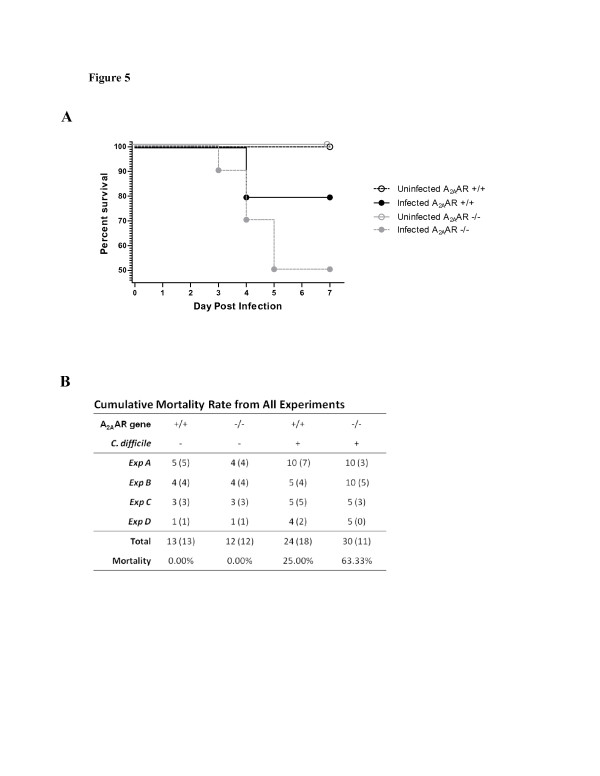
**Survival of A**_**2A**_**AR+/+or-/-Mice Infected with *C ***.***difficile. *A**. Survival Curve of *Exp B* showing 80% of the littermate wild-type vs. 50% of knockout mice surviving infection with VPI10463. *P* value for Log-rank (Mantel-Cox) Test is 0.1592; *P* value of Logrank test for trend is 0.0666. **B**. Cumulative mortality from all experiments (*Exp A*-*D*). Number of mice in each experiment (number of mice surviving infection).

To confirm the role of A_2A_AR in CDI, A_2A_AR knockout (KO) and wild-type littermate mice were infected with VPI10463. As seen in Figure 5A (*Exp B*), only 50% of infected KO mice survived compared to 80% of infected wild-type mice. Four similar experiments were performed to compare survival rates between infected A_2A_AR KOs and wild-type mice (Figure 5B). The overall survival rates in infected wild-type were consistently higher than infected KO mice (75% vs. 36.67%) suggesting that the absence of the A_2A_AR is detrimental during infection.

**Figure 6:**
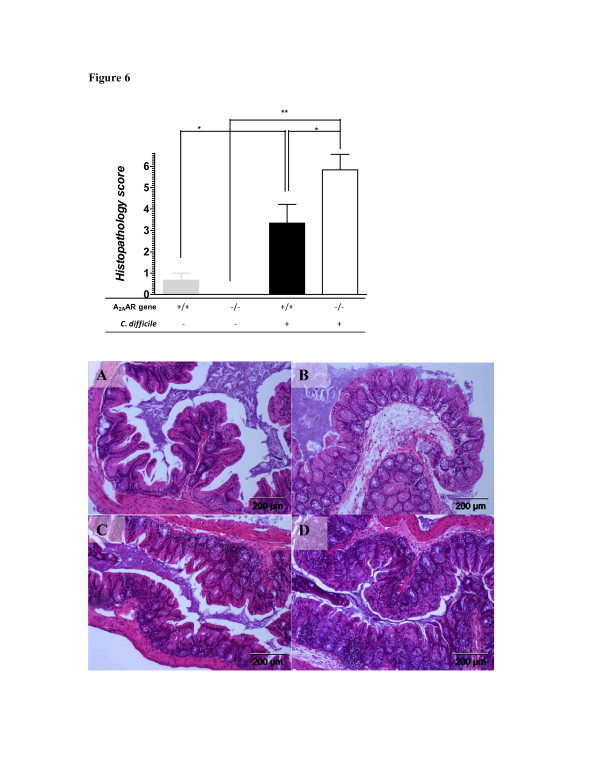
**Cecal Histopathology of A**_**2A**_**AR+/+or-/-Mice Infected with *C. difficile. *** Cecal tissues from surviving mice were obtained on the day 14 post-infection. Histopathological scores were from 3 cecal tissues per group. *p<0.05 and **p<.001 by One-way ANOVA with Bonferroni’s correction. Representative H&E stained tissues from uninfected (**A**) and infected (**B**) A_2A_AR +/+ and uninfected (**C**) and infected (**D**) A_2A_AR -/- mice.

Regardless of their genetic background, infected animals had significantly higher total cecal histopathology score than uninfected controls (Figure 6; *Exp B*). Moreover, cecal tissues from infected KO mice had higher histopathology scores than those from wild-type mice. Submucosal edema, mucosal thickness and inflammation were observed more in infected than uninfected cecal tissues (Figure 6A-D). The same parameters were worse in A_2A_AR gene deleted mice than wild types during infection confirming A_2A_AR’s protective role against infection-induced epithelial injury consistent with what was previously seen in toxin-induced enteritis [22].

### The absence of A_2A_AR activation altered the inflammatory response during CDI in mice

**Figure 7:**
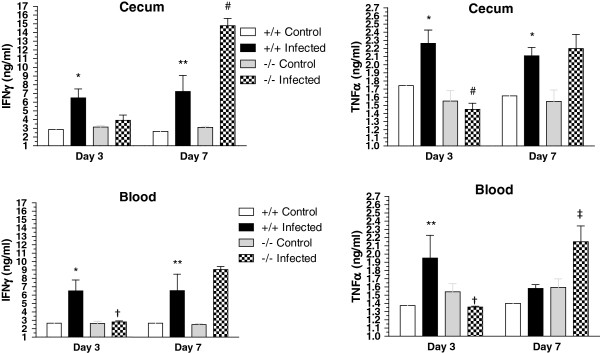
**Intestinal Tissue and Blood IFNγ and TNFα in *C. difficile *infected mice.** Cecal and plasma levels of IFNγ and TNFα were measured at days 3 and 7 post-infection in A_2A_AR -/- and A_2A_AR +/+ mice. Each bar from cecal levels represents 4 specimens (4 mice) per group. Each bar from blood levels represents 4 specimens per group except bars representing infected mice on day 3 (n=3/group). *P<0.05 or **p<0.01 for infected A_2A_AR +/+ vs. uninfected A_2A_AR +/+ in same day, ^†^p<0.05, ^‡^p<0.01 or ^#^p<0.001 for infected A_2A_AR -/- vs. infected A_2A_AR +/+ in same day; Two-way ANOVA with post-hoc Bonferroni’s correction.

To determine whether enhanced inflammation contributed to the greater epithelial injury and increased mortality in A_2A_AR-/- mice, cecal tissues and sera were assayed for IFNγ and TNFα. IFNγ and TNFα levels were increased during infection in wild-type mice at both days 3 and 7 post-infection (Figure 7). Unexpectedly, both cytokines were significantly depressed in A_2A_AR-/- compared to wild-type mice at day 3 suggesting less inflammation in the absence of A_2A_AR at the expected peak of infection. At day 7 post-infection, IFNγ and TNFα were significantly more elevated in cecal and blood, respectively, in A_2A_AR-/- mice than wild-type mice indicating that onset and resolution of inflammation were both delayed in the absence of A_2A_ARs. Furthermore, these results suggest that IFNγ may play a greater role in intestinal tissue injury while TNFα may be more involved in the systemic manifestations of infection.

## Discussion and conclusions

Vancomycin is the drug of choice for severe CDI [27,28]. However, its use has been associated with clinical recurrence of infection in up to 20% of cases, which has been attributed to antibiotic-induced changes of gut microbiota [29]. In the mouse model of infection, recurrence of disease and late mortality has also been observed to occur in 40-60% of mice treated with vancomycin [24,30]. Typically, mice treated with vancomycin remain well and only develop disease several days after cessation of the antibiotic. In our study, we demonstrated that the addition of A_2A_AR agonists during vancomycin treatment decreased recurrence and associated late mortality in mice infected with *C*. *difficile*. We then confirmed the role of A_2A_ARs in CDI by showing that the complete absence of A_2A_AR activation exacerbated disease and alteration of inflammatory cytokine expression.

Intestinal epithelial cells have a very limited capacity for *de novo* adenosine synthesis [31]. The villous cells can directly use absorbed dietary nucleosides but the cryptal cells depend on blood supply. Under normal adenosine homeostasis, extracellular adenosine concentrations can be lower than 1 μM [32,33]. In response to cellular damage, adenosine concentrations are quickly elevated 100-fold higher in inflamed intestine due to ATP and adenosine secretion in inflammatory and other cell types. Control of inflammation and injury is thought to be secondary to A_2A_AR activation in intestinal tissue reperfusion injury [34] or experimental colitis [35]. The A_2A_AR has been shown to inhibit neutrophil cytotoxic activities such as expression of β2-integrins [36], adhesion to endothelium [37], production of oxygen radicals [38,39], degranulation [40], and production of TNF-α [41]. Given intense intestinal inflammation, including neutrophilic tissue infiltration, noted in CDI in mouse [42], blockade or absence of A_2A_ARs would, then, be expected to result in aggravation of disease. Indeed, in our study, A_2A_AR knockout mice had worse colitis (even in mice surviving infection) and more deaths from *C*. *difficile* infection compared to wild-type littermates suggesting that endogenous adenosine provides protection against infection through the A_2A_AR. A_2A_AR agonists added to 3 days of vancomycin treatment improved survival, clinical scores, and cecum histopathology compared to vancomycin alone when treatment was started at least one day after the animals were infected. This benefit was probably mediated by the anti-inflammatory effects of A_2A_AR activation counteracting the pro-inflammatory effects of *C*. *difficile* toxins.

Previous studies have shown that A_2A_AR activation with ATL 313 or inhibition of adenosine deaminase attenuated *C*. *difficile* toxin A-induced ileitis in mice [20,21]. Myeloperoxidase activity, TNF-α production, cell death and histopathology were all noted to be reduced in ileal tissues treated with ATL 313. Recently, we showed that the A_2A_AR agonist, ATL370, decreased toxin A-induced secretion and epithelial injury in rabbit ileum and decreased KC (keratinocyte chemokine) and IL10 levels in mouse cecal tissues [22]. In the current study, we demonstrate that deletion of A_2A_ARs in mice, resulted in delayed but augmented expression of inflammatory cytokines, specifically IFNγ and TNFα during infection suggesting that initial inflammatory response is essential in controlling the disease. Indeed, the administration of ATL 370 alone resulted in greater and quicker mortality in most of the treated *C*. *difficile*-infected mice suggesting that A_2A_AR activation may inhibit the natural and beneficial early immune response initiated by infection. Furthermore, antibiotic treatment is essential to control clostridial burden and development of severe disease. This is consistent with what had been shown in a mouse model of sepsis where another A_2A_AR agonist, ATL 146e, was shown to reduce mortality in endotoxemia from LPS but not in *E*. *coli* septicemia unless antibiotic was also given [19]. It is possible that adenosine is utilized by bacteria to enhance virulence as observed in other pathogens [43,44]. Interestingly, although infected knockout mice had worse mortality than their infected wild-type littermates, 50% of these mice still survived in the absence of vancomycin treatment. Differences in intestinal flora (transgenic mice were bred in house) as well as other host factors may play a role in susceptibility to severe disease.

Our observation that A_2A_AR agonist alone (administered at the start of infection) worsened outcomes possibly due to inhibiting the natural immune response early on prompted us to investigate whether delaying A_2A_AR agonist treatment in addition to treating with vancomycin would improve outcomes. We showed that delaying the start of A_2A_AR agonist treatment post-infection was as good as starting A_2A_AR agonist at the same time as vancomycin administration suggesting benefit of A_2A_AR activation even at a later time during the course of infection and antibiotic treatment. While our study showed that antibiotic treatment is necessary to cure infection, we also demonstrated that overtreating with vancomycin may yield the worst outcomes. Reducing the antibiotic treatment to 2 days improved outcomes and adding A_2A_AR agonist to this regimen reduced the mortality associated with vancomycin treatment alone by 50%. These observations may have important ramifications if they translate to the clinical setting. If vancomycin (or antibiotic) treatment duration can be reduced by adding an A_2A_AR agonist, recovery of the gut microbiota may be facilitated and the recurrence of infection after antibiotic therapy may be improved considerably.

Although previous studies have shown that A_2A_AR activation confers significant protection against *C*. *difficile* toxin-induced ileitis and cecitis [20-22], protection against severe disease in the mouse model of infection seems limited. We recently reported that A_2B_AR inhibition or deletion, even in the absence of an anti-clostridial agent, improved outcome of CDI in mice [45]. During infection, the clostridial bacteria are localized in the lumen and mucosal surface of the intestinal tract. The A_2B_AR is the predominant adenosine receptor in human intestinal epithelial cells [46] and thus, may have a greater role than A_2A_AR in mediating local tissue inflammation in response to *C*. *difficile* infection. However, A_2A_AR activity may be critical in controlling inflammatory response from immune cells recruited to the intestinal tissues and/or circulating immune cells during severe disease. More studies are needed to elucidate the interactions between different adenosine receptor subtypes during enteric infection.

In conclusion, in a murine model of CDI, vancomycin treatment resulted to reduced weight loss and diarrhea during acute infection, but was associated with high recurrence and late-onset death, with overall mortality being worse than untreated infected controls. Deletion of A_2A_ARs in mice worsened disease from CDI. The administration of an A_2A_AR agonist reduced the late mortality associated with vancomycin use, suggesting a possible adjunctive benefit of A_2A_AR agonists in the management of CDI to prevent recurrent disease and improve survival.

## Competing interests

YL, GK, TCB, CAW and RLG have no competing interests. RAF and JR were formerly employed by Dogwood Pharmaceuticals, Inc. JL was a consultant for Dogwood Pharmaceuticals, Inc. RWS is a consultant for Dogwood Pharmaceuticals, Inc.

## Authors’ contributions

YL, RAF, TCR, GK conducted experiments. JR, RWS, JL, RLG, CAW participated in study design, critical review of data and manuscript. YL and CAW designed experiments, performed data analyses and drafted manuscript. All authors read and approved the final manuscript.

## Pre-publication history

The pre-publication history for this paper can be accessed here:

http://www.biomedcentral.com/1471-2334/12/342/prepub
